# New Year

**Published:** 1862-01

**Authors:** 


### NEW YEAR.

We take this opportunity of wishing our readers a "Happy New Year." In return we hope not only to receive a kind wish fer ourselves, but a *dollar* for the Buffalo Medical and Surgical Journal. If those of our friends who have not already done so, will remember this important item, in making up the sum total of our happiness and the existence and prosperity of the Journal, we will do our best to "pour blessings on their heads."

